# Caspase-11 Modulates Inflammation and Attenuates* Toxoplasma gondii* Pathogenesis

**DOI:** 10.1155/2016/9848263

**Published:** 2016-06-09

**Authors:** Sheryl L. Coutermarsh-Ott, John T. Doran, Caroline Campbell, Tere M. Williams, David S. Lindsay, Irving C. Allen

**Affiliations:** Department of Biomedical Sciences and Pathobiology, Virginia-Maryland College of Veterinary Medicine, Virginia Tech, Blacksburg, VA 24061, USA

## Abstract

*Toxoplasma gondii* is an obligate intracellular parasite that is the etiologic agent responsible for toxoplasmosis. Infection with* T. gondii* results in activation of nucleotide binding domain and leucine rich repeat containing receptors (NLRs). NLR activation leads to inflammasome formation, the activation of caspase-1, and the subsequent cleavage of IL-1*β* and IL-18. Recently, a noncanonical inflammasome has been characterized which functions through caspase-11 and appears to augment many biological functions previously considered to be dependent upon the canonical inflammasome. To better elucidate the function of this noncanonical inflammasome in toxoplasmosis, we utilized* Asc* ^−/−^ and* Casp11* ^−/−^ mice and infected these animals with* T. gondii*. Our data indicates that caspase-11 modulates the innate immune response to* T. gondii* through a mechanism which is distinct from that currently described for the canonical inflammasome.* Asc* ^−/−^ mice demonstrated increased disease pathogenesis during the acute phase of* T. gondii* infection, whereas* Casp11* ^−/−^ mice demonstrated significantly attenuated disease pathogenesis and reduced inflammation. This attenuated host response was associated with reduced local and systemic cytokine production, including diminished IL-1*β*. During the chronic phase of infection, caspase-11 deficiency resulted in increased neuroinflammation and tissue cyst burden in the brain. Together, our data suggest that caspase-11 functions to protect the host by enhancing inflammation during the early phase of infection in an effort to minimize disease pathogenesis during later stages of toxoplasmosis.

## 1. Introduction

*Toxoplasma gondii* is an intracellular apicomplexan parasite that can infect a variety of vertebrate hosts including humans and domestic animals. Transmission can occur through food or drinking water contaminated with infective oocysts, ingestion of meat infected with tissue cysts, or transplacentally from the mother to the fetus. Infection rates in humans are high but clinical disease is most problematic in immunocompromised individuals or when the infection is congenital. It is well established that the Toll-like receptor (TLR) family of pattern recognition receptors (PRRs) plays a critical role in host defense against* T. gondii*. The TLR associated adaptor protein myeloid differentiation primary response gene 88 (MyD88) has been found to be essential in the production of proinflammatory cytokines including IL-12 and IFN-*γ* [1–3]. Likewise, roles have been described for TLR-2, TLR-4, TLR-5, TLR-9, TLR-11, and TLR-12 [4–11].

In addition to the TLRs, other families of PRRs have been shown to play a role in the innate immune response against* T. gondii*. Recent studies have focused on members of the nucleotide binding domain and leucine rich repeat containing family of receptors (also referred to as NLRs). NLRs are cytosolic PRRs which are important modulators of inflammation through their regulation of the proinflammatory cytokines IL-1*β* and IL-18, as well as their role in the proinflammatory form of cell death termed pyroptosis [12]. Once a ligand binds the protein receptor, there is oligomerization with procaspase-1 and the adaptor molecule apoptosis-associated speck-like protein containing carboxy-terminal caspase activation and recruitment domain (ASC) to form a multimeric protein complex termed the inflammasome. This process cleaves cytosolic procaspase-1 into its active form, which then cleaves cytosolic pro-IL-1*β* and pro-IL-18 into their active forms. A diverse subgroup of NLRs have been identified as forming inflammasomes following the sensing of specific signals associated with intracellular pathogens [13]. The inflammasome forming NLR NLRP1 (nucleotide binding domain and leucine rich repeat containing receptor P1) has previously been identified as an essential sensor of* T. gondii* in rodents and mutations in* NLRP1 *have been shown to confer susceptibility for human congenital toxoplasmosis [14–18]. Likewise, the inflammasome forming NLR NLRP3 has also been shown to confer host protection following* T. gondii* infection [17].

Recently, a noncanonical inflammasome has been identified and characterized [19]. This noncanonical inflammasome is responsible for recognizing intracellular lipopolysaccharide (LPS), as well as recognizing and regulating the host immune response to* Escherichia coli*,* Citrobacter rodentium*, and* Vibrio cholera* [19]. Activation of the noncanonical inflammasome appears to occur during acute inflammatory conditions, such as sepsis, and activation results in IL-1*β* and IL-18 cleavage through a canonical inflammasome-dependent mechanism [19]. Activation of the noncanonical inflammasome can also lead to pyroptosis; however, this occurs through a mechanism that is independent of the canonical inflammasome [19]. Unlike the canonical inflammasome, there is currently a paucity of data pertaining to noncanonical inflammasome activation and function. For example, it has recently been shown that LPS can directly bind caspase-11, which is a critical caspase, to activate the noncanonical inflammasome [20]. However, it is not clear if other pathogen associated molecular patterns (PAMPs) can stimulate this pathway or if noncanonical signaling plays any role in host-pathogen responses that are not associated with bacteria.

In the present study, we investigated the role of caspase-11, which is an essential component of the noncanonical inflammasome, in the pathogenesis of* T. gondii*. We hypothesized that caspase-11 would significantly contribute to the host innate immune response following* T. gondii* infection, particularly during the acute phase, which is characterized by robust inflammation. Our data show that IL-1*β* levels are partly dependent on caspase-11* ex vivo* in macrophages and* in vivo* in mice.* Casp11* ^−/−^ animals appear to be relatively resistant to* T. gondii* as they show significantly attenuated changes in morbidity and mortality and reduced inflammation during the early phase of disease. This is in stark contrast to the increased sensitivity observed in the* Asc* ^−/−^ mice also evaluated in this study. Unfortunately, these protective effects do not extend into later phases of the disease as* Casp11* ^−/−^ mice develop significantly increased neuroinflammation and brain tissue cysts, likely due to the attenuated local inflammatory response during the acute phase. Thus, our findings establish a role for caspase-11 beyond the host immune response to bacteria and demonstrate a critical role in the host-pathogen response following* T. gondii* infection.

## 2. Materials and Methods

### 2.1. Experimental Animals

All studies were conducted in accordance with Institutional Animal Care and Use Committee (IACUC) guidelines and according to the institutionally approved animal protocol. The generation and characterization of* Casp11* ^−/−^ and* Asc* ^−/−^ mice have previously been described [19, 21]. All experiments were conducted with 6–8-week-old, female mice backcrossed for at least 12 generations onto the C57Bl/6 background. Mice were injected intraperitoneally with 1,000* T. gondii* tachyzoites (strain ME49) diluted in 400 *μ*L phosphate-buffered saline (PBS). Morbidity and mortality were monitored daily for 25 days after inoculation (d.p.i.). Weight loss and clinical scores were assessed daily following* T. gondii* inoculation. The clinical score is derived from individual assessments of weight loss, body condition, behavior, and gait, each individually scored on a scale of 0–5. Individual scores are combined and averaged to generate the composite clinical score. Mice were euthanized at day 15 after inoculation to evaluate acute (or early)* T. gondii* infection or at day 25 after inoculation to evaluate chronic (or late)* T. gondii* infection. At euthanasia, whole blood was collected via cardiac puncture and tissues were collected for further processing.

### 2.2. Assessments of Inflammation

At harvest, samples of brain, lung, heart, liver, spleen, and intestine were collected and either stored at −80°C for protein analysis or placed into 10% buffered formalin for histopathology. Peritoneal lavage fluid (PLF) was taken prior to organ collection. Briefly, the skin overlying the abdominal cavity was incised and reflected to reveal an intact peritoneum. A 27-gauge needle was inserted and the abdomen was flushed with 5 mL of sterile PBS. The samples were spun down and cell supernatants utilized for cytokine analysis. Cell pellets were resuspended and evaluated on a hemacytometer for total nucleated cell counts. Additionally, cytospin preparations were made and evaluated with Dif-Quik for differential cell counts.

### 2.3. Histopathologic Examination

Formalin-fixed tissues were routinely processed for histopathology. The paraffin-embedded tissues were sectioned at 5 *μ*m and prepared for hematoxylin and eosin (H&E) staining. H&E stained sections were evaluated by a board-certified veterinary pathologist (S.C.O.). Composite scores for brain inflammation were determined by the numbers of inflammatory cells within the leptomeninges (0–2) as well as within the parenchyma itself (0–3). For the leptomeninges, scores were given as follows: 0, no inflammatory cells identified, 1, less than 3 layers of inflammatory cells identified, or 2, greater than or equal to 3 layers of inflammatory cells identified. For the parenchyma, scores were given as follows: 0, no inflammatory cells identified, 1, less than 3 layers of inflammatory cells identified in perivascular spaces only, 2, greater than 3 layers of inflammatory cells identified in perivascular spaces only, or 3, inflammatory cells identified in perivascular spaces as well as within the neuroparenchyma.* T. gondii* cysts were identified as present or absent.

### 2.4. Bone Marrow Derived Macrophage Isolation and Evaluation

Bone marrow derived macrophages (BMDMs) were isolated from the femurs of C57Bl/6,* Casp11* ^−/−^, and* Asc* ^−/−^ mice using standard procedures. The cells were cultured in Dulbecco's modified Eagle's medium with 10% fetal bovine serum, 20% L929-conditioned cell culture supernatant, 1x L-glutamine, and 1x nonessential amino acids for 7 days. BMDMs with or without LPS priming (100 ng/mL for 30 minutes) were infected with Me49 tachyzoites (Moiety of Infection = 1) for 24 hours. Supernatants were removed for cytokine measurements.

### 2.5. Cytokine Assessments

Cytokine analysis on cell-free supernatants from PLF, BMDM supernatants, and tissue homogenates as well as serum was performed using standard enzyme-linked immunosorbent assay (ELISA) kits (BD Biosciences). All results were normalized per weight of tissue or volume of original sample.

### 2.6. Statistical Analysis

We utilized GraphPad Prism 5 statistical software to conduct Analysis Of Variance (ANOVA) followed by either Tukey-Kramer honest significant difference or Newman-Keuls posttest to evaluate statistical significance for multiple comparisons. Single data point comparisons were evaluated by Student's two-tailed *t*-test. Group survival was assessed utilizing the Kaplan-Meier test. All data are presented as the mean ± the standard error of the mean (SEM) and in all cases a *p* value less than 0.05 was considered statistically significant.

## 3. Results

### 3.1. The Canonical NLR Inflammasome and Caspase-11 Are Associated with IL-1*β* Production in Murine Macrophages following* Toxoplasma gondii* Infection

**Figure 1:**
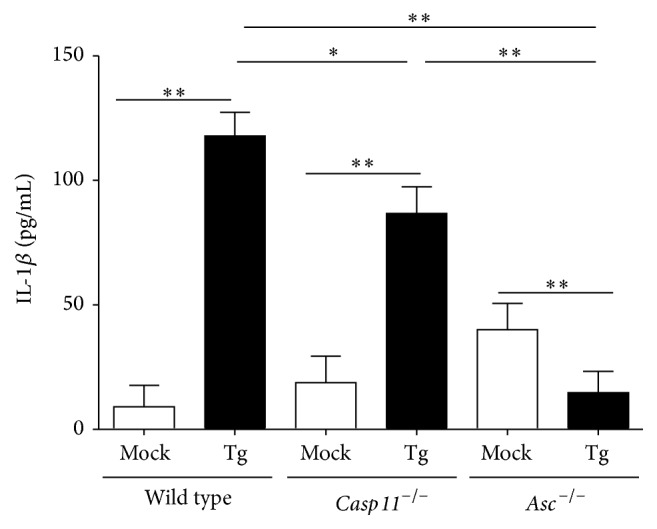
IL-1*β* levels are ablated in macrophages harvested from* Casp11* ^−/−^ and* Asc* ^−/−^ mice following* ex vivo* infection with* Toxoplasma gondii*. Bone marrow derived macrophages were primed with 100 ng/mL of LPS for 30 mins prior to stimulation with either* Toxoplasma gondii* Me49 (MOI = 1) or vehicle for 24 hrs. IL-1*β* levels in cell-free supernatants were quantified using ELISA. ^*∗*^ *p* < 0.05; ^*∗∗*^ *p* < 0.01. Data shown are representative of 3 independent experiments utilizing 2 mice per genotype.

While the role of the canonical NLR inflammasome and caspase-1 in* T. gondii* infection has been characterized, the contribution of the noncanonical inflammasome and caspase-11 has yet to be evaluated [14–18]. To determine if caspase-11 functions in the host-parasite response, primary BMDMs were isolated from wild type,* Asc* ^−/−^, and* Casp11* ^−/−^ mice, as previously described [22]. Previous studies have shown that canonical inflammasome activation and secretion of IL-1*β* by macrophages following exposure to* T. gondii* Me49 are dependent upon LPS priming and parasite internalization and occur independent of cell death [17]. To evaluate these findings in the* Casp11* ^−/−^ macrophages, cells were primed with 100 ng/mL of LPS for 30 minutes followed by exposure to* T. gondii* Me49 tachyzoites (MOI = 1) or vehicle.* T. gondii* infection resulted in a significant increase in IL-1*β* in wild type macrophages compared to LPS primed mock infected macrophages (Figure 1). Consistent with the previously demonstrated essential nature of the canonical NLR inflammasome in sensing* T. gondii*, IL-1*β* levels were markedly reduced in macrophages isolated from* Asc* ^−/−^ mice (Figure 1).* Casp11* ^−/−^ macrophages infected with* T. gondii*, interestingly, displayed intermediate levels of IL-1*β* when compared to infected wild type and infected* Asc* ^−/−^ macrophages (Figure 1). The attenuation in IL-1*β* levels, rather than full ablation, observed in the* Casp11* ^−/−^ macrophages suggests that caspase-11 and the noncanonical inflammasome likely function to augment the activity of the canonical inflammasome. This is consistent with prior observations that suggest a synergistic model for caspase-11 function in bacteria sensing, where the noncanonical inflammasome complements canonical inflammasome signaling and IL-1*β* maturation during acute inflammation [19, 23–25].

### 3.2. *In Vivo* Caspase-11 Increases* Toxoplasma gondii* Pathogenesis in the Early Stages of Disease

**Figure 2:**
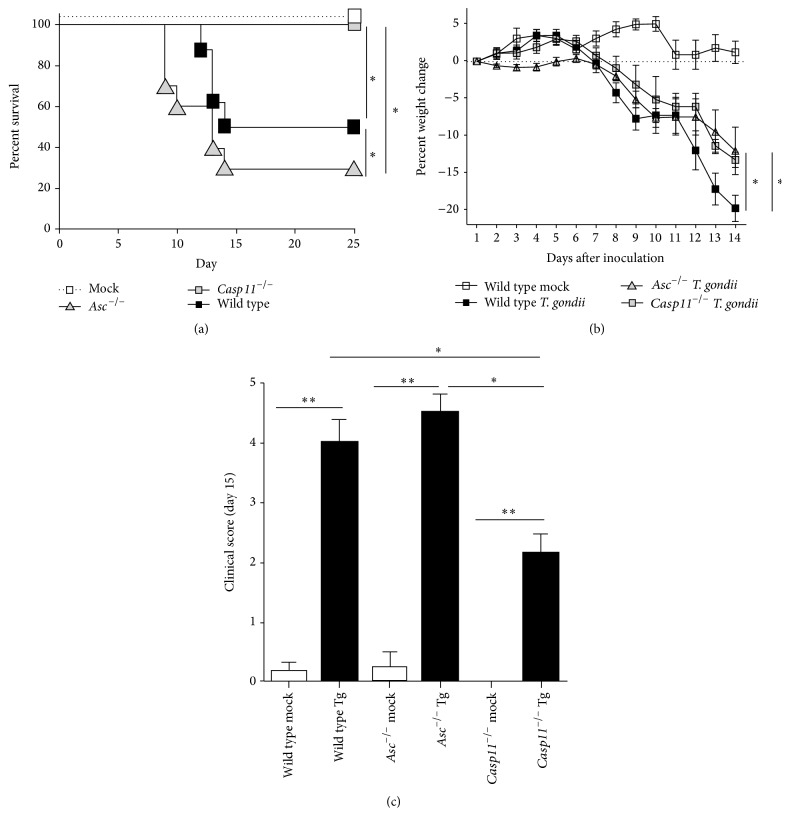
*Casp11* ^−/−^ mice exhibit reduced susceptibility to* Toxoplasma gondii* infection. Wild type,* Casp11* ^−/−^, and* Asc* ^−/−^ mice were infected with 1,000* Toxoplasma gondii* Me49 tachyzoites via i.p. administration. Morbidity and mortality were monitored daily. (a) Kaplan-Meier plot of WT,* Asc* ^−/−^, and* Casp11* ^−/−^ mice survival. Mice were considered moribund when weight loss was sustained at or below −15% from baseline and/or clinical parameters necessitated euthanasia. (b) Weight loss was evaluated daily and the percent change from baseline for each animal was calculated. Data shown reflect 1–14 days after inoculation due to the significantly reduced survival of the wild type and* Asc* ^−/−^ mice at day 15. (c)* Casp11* ^−/−^ mice demonstrate significantly attenuated clinical parameters associated with disease progression. The clinical score is a composite of scores associated with weight loss, clinical condition, and behavior. ^*∗*^ *p* < 0.05. Wild type mock, *n* = 9;* Asc* ^−/−^ mock, *n* = 9;* Casp11* ^−/−^ mock, *n* = 6; wild type Tg, *n* = 18;* Asc* ^−/−^ Tg, *n* = 18;* Casp11* ^−/−^ Tg, *n* = 13. Data shown are representative of 3 independent studies.

Activation of the canonical NLRP1 and NLRP3 inflammasomes controls* T. gondii* proliferation and minimizes disease pathogenesis [16, 17]. This conclusion is based on prior studies, which have revealed that* Nlrp1* ^−/−^,* Nlrp3* ^−/−^,* Casp1/11* ^−/−^, and* Asc* ^−/−^ mice are highly susceptible to acute toxoplasmosis [16, 17]. To evaluate the contribution of caspase-11 in disease pathogenesis, wild type,* Casp11* ^−/−^, and* Asc* ^−/−^ mice were infected with 1,000* T. gondii* Me49 tachyzoites via intraperitoneal (i.p.) injection. Survival, weight change, and clinical parameters associated with toxoplasmosis were assessed daily (Figure 2). During early stages of the disease, typically characterized by tachyzoite proliferation and acute inflammation, we observed a significant decrease in survival of both wild type and* Asc* ^−/−^ mice compared to mock injected animals (Figure 2(a)). Fifty percent of the wild type mice required euthanasia by 14 d.p.i. In the* Asc* ^−/−^ mice, we observed a significant decrease in survival beginning at 9 d.p.i. and extending through 14 d.p.i. Ultimately, 70% of the* Asc* ^−/−^ mice required euthanasia due to acute toxoplasmosis (Figure 2(a)). Conversely, no* Casp11* ^−/−^ mice required euthanasia and all of the animals survived (Figure 2(a)). These findings were also reflected in the weight loss and clinical score data. All animals inoculated with* T. gondii* demonstrated significantly increased weight loss (Figure 2(b)). Wild type animals demonstrated a consistent decrease in body weight between 8 and 14 d.p.i., which was significantly greater than the weight loss observed for either the* Asc* ^−/−^ or* Casp11* ^−/−^ mice (Figure 2(b)). While weight loss was more rapid in the wild type animals, the* Asc* ^−/−^ mice demonstrated higher clinical scores reflecting more severe disease presentation (Figure 2(c)). Assessments of clinical parameters associated with toxoplasmosis revealed that disease progression was indeed severe in both wild type and* Asc* ^−/−^ mice while it was significantly attenuated in the* Casp11* ^−/−^ animals (Figure 2(c)). Weight loss and clinical parameters associated with disease were monitored through day 25 in the surviving animals (data not shown). While these parameters remained significantly reduced (5–10% decrease) in all genotypes compared to the mock treated animals, all of the animals that survived the acute phase of* T. gondii* infection demonstrated a significant improvement in morbidity and mortality throughout the remainder of the study (data not shown). Together, these data confirm previous findings by demonstrating the essential role of the canonical inflammasome in maintaining host resistance to* T. gondii*. In addition, these findings also reveal a unique contribution for caspase-11 which appears to be distinct from ASC and suggests that caspase-11 actually contributes to disease severity during the acute phase of* T. gondii *infection.

### 3.3. Severe Toxoplasmosis Was Observed during the Acute Phase of* Toxoplasma gondii* Infection That Resolved during the Chronic Phase in All Surviving Animals

**Figure 3:**
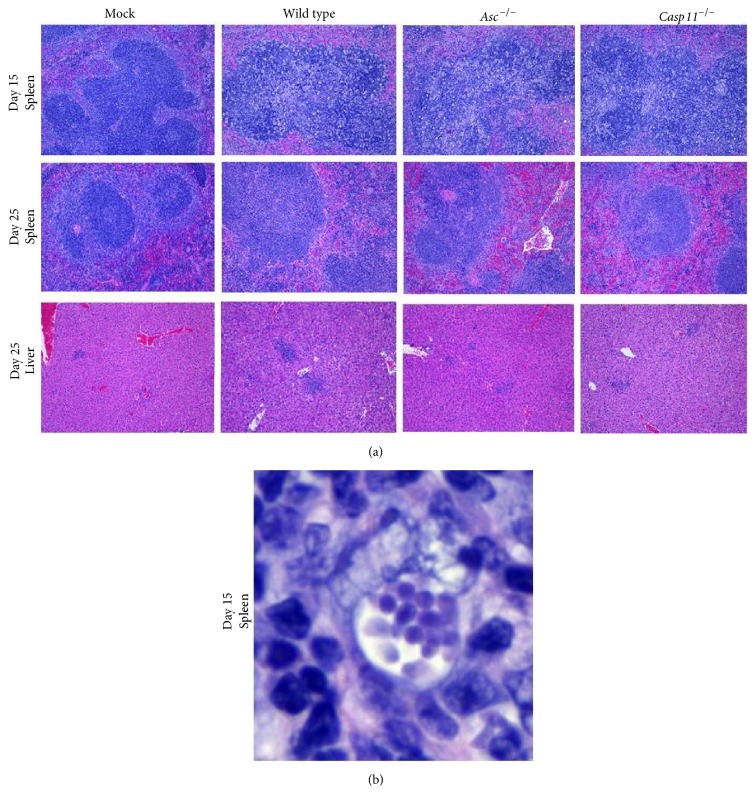
*Toxoplasma gondii* tachyzoites induced severe toxoplasmosis which resolved during the chronic phase of disease in all surviving animals. Wild type,* Casp11* ^−/−^, and* Asc* ^−/−^ mice were infected with 1,000* Toxoplasma gondii* Me49 tachyzoites via i.p. administration and necropsied at 15 and 25 days after inoculation (d.p.i.). (a) Tachyzoite injection resulted in significantly increased spleen histopathology at 15 d.p.i. in all animals regardless of genotype. By 25 d.p.i., spleen histopathology was markedly improved with only minimal evidence of prior disease for all inoculated genotypes. Mild-to-moderate inflammation was observed in the livers of all infected mice. 20x magnification. (b)* T. gondii* tachyzoites were found in high concentrations in the spleen of all inoculated animals at 15 d.p.i. with no significant difference noted between genotypes. 100x magnification. Wild type mock, *n* = 9;* Asc* ^−/−^ mock, *n* = 9;* Casp11* ^−/−^ mock, *n* = 6; wild type Tg, *n* = 18;* Asc* ^−/−^ Tg, *n* = 18;* Casp11* ^−/−^ Tg, *n* = 13. Data shown are representative of histopathology evaluated over the course of 3 independent studies.

Previous studies have shown that* Casp1/11* ^−/−^ mice are highly susceptible to* T. gondii* infection in part due to failure to control parasite load in the peritoneal cavity [16, 17]. These prior studies utilized green fluorescent protein-luciferase (GFP-LUC) expressing tachyzoites and whole body imaging to evaluate parasite load. To investigate these findings in our studies utilizing* Asc* ^−/−^ and* Casp11* ^−/−^ mice, we evaluated histopathologic sections of peritoneal organs to directly assess disease pathogenesis (Figure 3). All histopathology was evaluated by a board certified veterinary pathologist (S.C.O). At 15 days after infection, we detected robust signs of infection and inflammation. Spleens from mice harvested at Day 15 were characterized by expansion of the lymphoid follicles by large numbers of macrophages occasionally containing intracellular tachyzoites as well as moderately increased numbers of lymphocytes and plasma cells. Lymphocytolysis was a prominent feature in the spleen and numerous animals exhibited pronounced accumulations of fibrin, cellular debris, and intrahistiocytic and extracellular tachyzoites on the serosal surface (Figures 3(a) and 3(b)). No significant difference was observed between wild type,* Asc* ^−/−^, or* Casp11* ^−/−^ animals. Spleens from mice harvested at day 25 exhibited milder evidence of disease characterized by significantly less inflammation and no evidence of intracellular or extracellular tachyzoites (Figure 3(a)). Additionally, by day 25, all infected animals had developed mild evidence of inflammation in the liver as well (Figure 3(a)). Additionally, we did detect a significant tachyzoite burden and moderate-to-severe inflammation in the lungs in infected animals harvested at day 25 (data not shown). However, our analysis revealed that there were no significant differences in histopathology or parasite burden between the wild type,* Asc* ^−/−^, and* Casp11* ^−/−^ mice in any of the abdominal or thoracic organs evaluated. This is in contrast to the prior study that suggested increased parasite replication in the absence of the canonical inflammasome [17]. The differences observed between the prior studies and the current data can be reconciled by considering methodological differences between the models as well as temporal and spatial differences in disease pathogenesis. Both prior studies utilized bioluminescence as an indirect assessment of disease pathogenesis [16, 17], whereas the current study utilized histopathological evaluation. Histopathological assessments are a more direct method of assessment and provide higher resolution associated with parasite localization. However, this approach is less quantitative compared to the bioluminescence approach. Likewise, the prior study evaluating* Asc* ^−/−^ mice focused on earlier time points (days 5 and 7), which may reflect the peak in tachyzoite proliferation [17]. Meanwhile, the current study evaluated day 15 which represented the peak in morbidity and mortality and may better reflect the peak in the host immune response.

### 3.4. Inflammation during the Acute Phase of* Toxoplasma gondii *Infection Is Increased by Caspase-11

**Figure 4:**
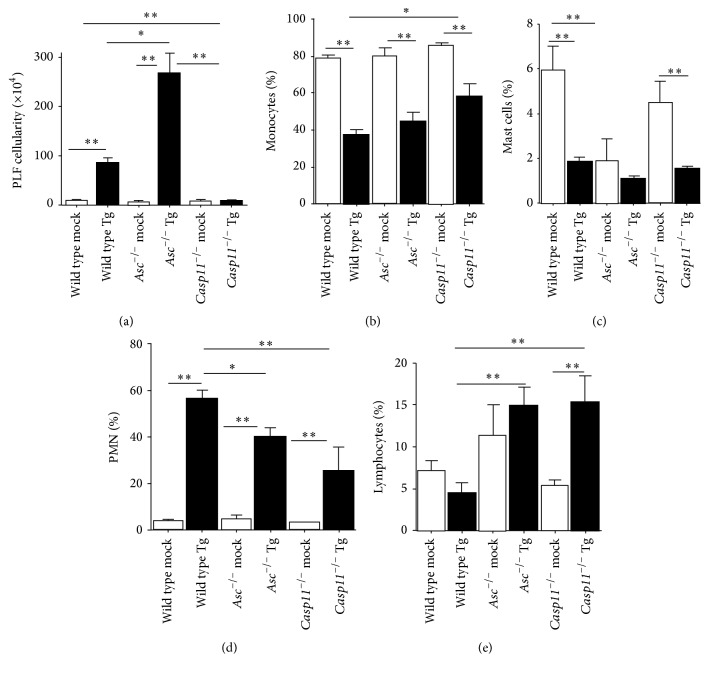
Peritoneal inflammation was significantly attenuated in* Casp11* ^−/−^ mice. Wild type,* Casp11* ^−/−^, and* Asc* ^−/−^ mice were infected with 1,000* Toxoplasma gondii* Me49 tachyzoites via i.p. administration and peritoneal lavage fluid (PLF) was collected 15 days after inoculation. (a) Total PLF cellularity was determined for each treatment group using trypan blue staining and a hemacytometer. (b–e) PLF leukocyte populations were determined following differential staining of cytospun samples. The percentages of monocytes (b), mast cells (c), polymorphonuclear cells (neutrophils and eosinophils) (d), and lymphocytes (e) were determined for each animal. ^*∗*^ *p* < 0.05; ^*∗∗*^ *p* < 0.01. Data shown are representative of histopathology evaluated over the course of 3 independent studies. Wild type mock, *n* = 9;* Asc* ^−/−^ mock, *n* = 9;* Casp11* ^−/−^ mock, *n* = 6; wild type Tg, *n* = 18;* Asc* ^−/−^ Tg, *n* = 18;* Casp11* ^−/−^ Tg, *n* = 13. Data shown are representative of 3 independent studies.

*T. gondii* infection results in the rapid and acute induction of the innate immune response, which results in significant leukocyte migration. To evaluate this response in the absence of ASC and caspase-11, we collected PLF from animals 15 d.p.i. The total cellularity of each animal was evaluated using trypan blue staining and counted on a hemacytometer.* T. gondii* infection resulted in a significant increase in leukocytes in the peritoneal cavity in wild type mice (Figure 4(a)). We observed a significant increase in PLF cellularity in the* Asc* ^−/−^ mice compared to the wild type animals (Figure 4(a)). These findings are consistent with a loss of immune system homeostasis and increased overall inflammation in these animals as described in other models of acute disease [25–27]. Unlike* Asc* ^−/−^ mice, we observed a significant attenuation in PLF cellularity in* Casp11* ^−/−^ animals compared to wild type mice (Figure 4(a)). Overzealous inflammation is a major contributing factor to increased morbidity and reduced survival during acute disease. Thus, the significantly attenuated leukocyte infiltration observed in* Casp11* ^−/−^ mice in the peritoneal cavity is consistent with the improved survival and reduced clinical features observed in these animals. In order to better characterize the infiltrating leukocytes, cytospins were generated from each PLF sample and differential staining was utilized to determine the specific cell populations associated with the immune response for each genotype. The cellular composition of the PLF was significantly altered 15 d.p.i. with* T. gondii* (Figures 4(b)–4(e)). Monocyte derived cells represented the dominant cellular population in all mock treated animals (Figure 4(b)). However, following* T. gondii* infection, we observed a significant decrease in the percentage of monocytes and a significant increase in polymorphonuclear (PMN) cells in the PLF (Figures 4(b) and 4(d)). In both* Asc* ^−/−^ and* Casp11* ^−/−^ mice, we observed significant decreases in the PMN cell population and significant increases in the lymphocyte population compared to the wild type mice (Figures 4(d) and 4(e)). We also observed a significant increase in the percentage of monocytes in* Casp11* ^−/−^ mice compared to wild type mice (Figure 4(b)). Interestingly, we observed a significant decrease in the mast cell populations in both mock and* T. gondii* infected* Asc* ^−/−^ mice (Figure 4(c)). Decreased mast cell populations have not been reported in* Asc* ^−/−^ mice and, if confirmed, may represent an interesting direction for future studies. In the context of* T. gondii* infection, reduced numbers of mast cells have been correlated to increased disease pathogenesis [28] and may underlie the increased disease severity in* Asc* ^−/−^ mice reported here and in prior studies [16, 17]. Together, these data reflect significant differences in the overall immune response between* Asc* ^−/−^ mice and* Casp11* ^−/−^ animals, whereas the composition of the cells being recruited to the peritoneal cavity is similar between the two mouse strains during acute toxoplasmosis.

### 3.5. Local and Systemic Levels of Proinflammatory Cytokines Are Significantly Attenuated during the Acute Phase of* Toxoplasma gondii* Infection in the Absence of Caspase-11

**Figure 5:**
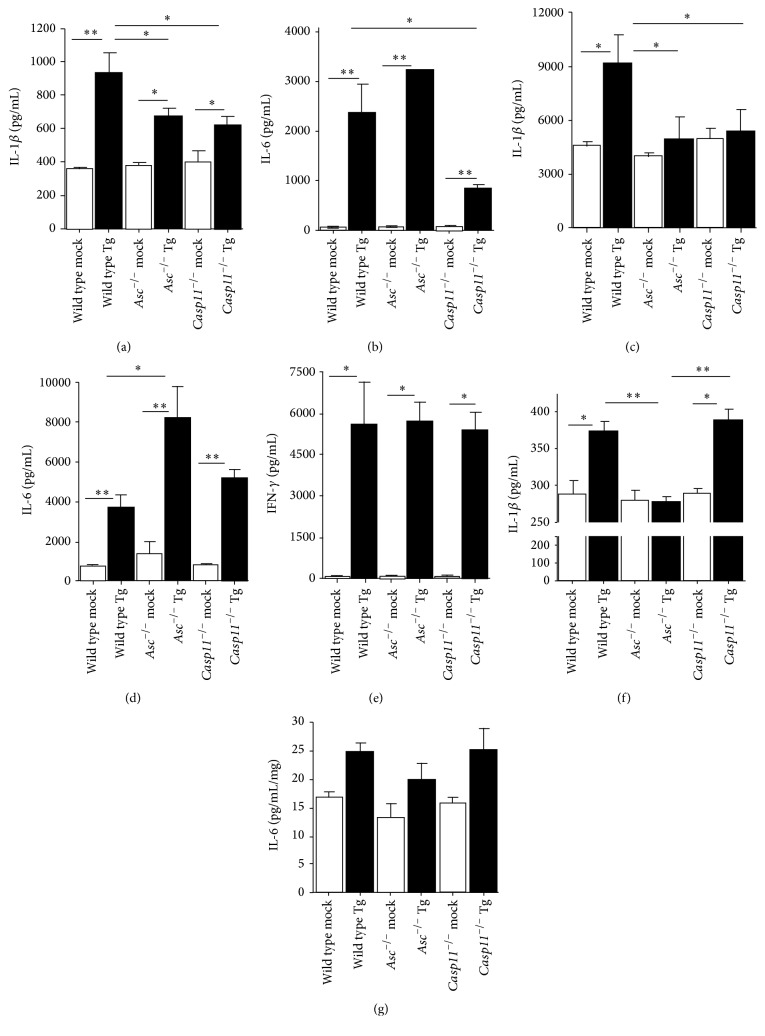
Local and systemic levels of IL-1*β* and IL-6 were significantly attenuated in* Casp11* ^−/−^ mice following infection with* Toxoplasma gondii*. Whole blood, peritoneal lavage fluid (PLF), and brains were collected from wild type,* Casp11* ^−/−^, and* Asc* ^−/−^ mice 15 days after inoculation with* Toxoplasma gondii* Me49 (a–d). IL-1*β* and IL-6 levels were determined in (a, b) serum and (c, d) cell-free PLF supernatant by ELISA. (e) IFN-*γ* levels were also evaluated in the cell-free PLF supernatant. (f, g) Brain samples were weighed and homogenized, and IL-1*β* and IL-6 levels were determined using the resultant cell-free supernatants. ^*∗*^ *p* < 0.05; ^*∗∗*^ *p* < 0.01. Wild type mock, *n* = 9;* Asc* ^−/−^ mock, *n* = 9;* Casp11* ^−/−^ mock, *n* = 6; wild type Tg, *n* = 18;* Asc* ^−/−^ Tg, *n* = 18;* Casp11* ^−/−^ Tg, *n* = 13. Data shown are representative of 3 independent studies.

IL-1*β* production following* T. gondii* infection has been shown to be dependent upon canonical NLRP1 and NLRP3 inflammasome activation [16, 17]. To evaluate the contribution of caspase-11 in this process, we evaluated systemic and local cytokine levels in the serum and PLF, respectively, from our wild type,* Asc* ^−/−^, and* Casp11* ^−/−^ mice.* T. gondii* infection resulted in a significant increase in both systemic and local levels of both IL-1*β* and IL-6 in the wild type animals 15 d.p.i. (Figures 5(a)–5(d)). Following infection, IL-1*β* was significantly attenuated in both serum and PLF from* Asc* ^−/−^ mice compared to the wild type animals (Figures 5(a) and 5(c)). This appears to be specific to IL-1*β* as IL-6 levels were significantly higher in* Asc* ^−/−^ mice compared to the wild type animals (Figures 5(b) and 5(d)). In addition to our findings in* Asc* ^−/−^ mice, we also observed a significant attenuation in both local and systemic IL-1*β* levels during acute* T. gondii* infection in the* Casp11* ^−/−^ animals (Figures 5(a) and 5(c)). However, unlike the data reported for mice lacking ASC, we also observed a significant attenuation in systemic IL-6 levels in the serum from the* Casp11* ^−/−^ mice compared to the wild type mice (Figure 5(b)). Local levels of IL-6 were significantly increased in the PLF from the* Casp11* ^−/−^ mice, similar to wild type levels, following* T. gondii* infection (Figure 5(d)). The reduction in systemic IL-6 likely reflects the overall improvement in morbidity and mortality and attenuated immune response observed in the* Casp11* ^−/−^ animals compared to the wild type and* Asc* ^−/−^ mice. In addition to IL-1*β* and IL-6, we also evaluated IFN-*γ* levels in the PLF (Figure 5(e)). IFN-*γ* is a critical cytokine that plays an essential role in controlling* T. gondii* proliferation and host protection [3, 29]. IFN-*γ* levels were significantly increased 15 d.p.i. in all of the infected mice compared to the mock treated animals. However, no significant difference in IFN-*γ* was detected between the different genotypes (Figure 5(e)). In addition to local and systemic cytokine responses, we also evaluated IL-1*β* and IL-6 levels in the brain at 15 d.p.i. The brain is one of the primary target organs of* T. gondii*. During the acute phase of infection, we observed a significant increase in IL-1*β* in brains collected from both wild type and* Casp11* ^−/−^ mice compared to the mock treated animals, with no differences between genotypes (Figure 5(f)). However, IL-1*β* was significantly attenuated in* Asc* ^−/−^ mice compared to either wild type or* Casp11* ^−/−^ animals (Figure 5(f)). It should be noted that the background levels of IL-1*β* were significantly increased in the brains from all animals regardless of genotype or treatment (Figure 5(f)). This is consistent with previous data that reported increased pro-IL-1*β* transcription levels in the brain at baseline [30]. In general, we have found this to be typical in tissue homogenates, which often include high levels of pro-IL-1*β* which is recognized by the ELISA based assays used in the current study. IL-6 was also evaluated in these samples. While we observed a slight increase in IL-6 in all infected mice, the overall levels of this cytokine were very low in the brain at this stage of* T. gondii* infection (Figure 5(g)). Together, these data are consistent with the prior studies evaluating ASC function in the acute stages of* T. gondii* infection and confirm the previous observations that IL-1*β* processing is deficient in the absence of canonical inflammasome signaling [16, 17]. Prior studies have shown that the noncanonical inflammasome augments canonical inflammasome function and the maturation of IL-1*β* during acute inflammation [31]. The data shown here are consistent with this previously described mechanism and further suggest that caspase-11, and more broadly the noncanonical inflammasome, likely functions through similar mechanisms as those previously reported for sepsis and acute bacteremia to modulate the host-parasite response to* T. gondii* [19].

### 3.6. Caspase-11 Mediated Inflammation during Acute* Toxoplasma gondii* Infection Results in Decreased Neuroinflammation during the Chronic Phase

**Figure 6:**
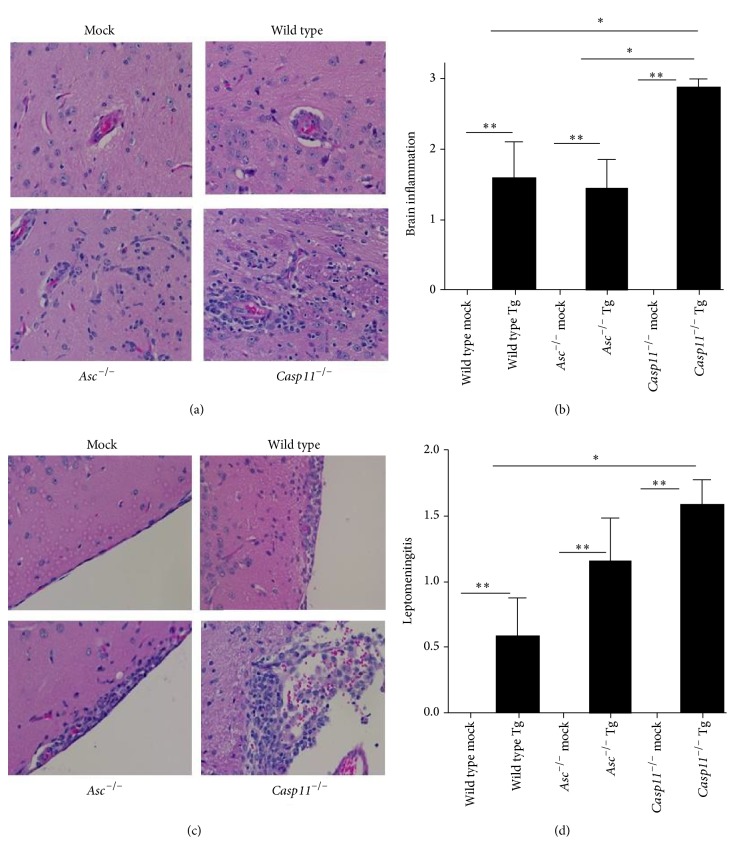
*Toxoplasma gondii* infection resulted in increased brain inflammation in* Casp11* ^−/−^ mice during late stages of disease. Wild type,* Casp11* ^−/−^, and* Asc* ^−/−^ mice were infected with 1,000* Toxoplasma gondii* Me49 tachyzoites via i.p. administration and brains were evaluated 25 days after inoculation in surviving mice. (a) Histopathologic assessments of H&E stained sections revealed increased inflammation in the brains from all animals infected with* T. gondii*. 20x magnification. (b) Parameters associated with neuroinflammation were assessed and scored for each animal. Composite scores for each animal were averaged to generate a semiquantitative brain inflammation score. (c) Leptomeningitis was a predominate feature in all* T. gondii* infected animals. 20x magnification. (d) Histopathology scoring revealed significant increases in leptomeningitis in* Casp11* ^−/−^ mice. ^*∗*^ *p* < 0.05; ^*∗∗*^ *p* < 0.01. For all mock treated animals, *n* = 3. For all* T. gondii* inoculated animals, *n* = 7. Data shown are representative of 2 independent studies.

The chronic phase of* T. gondii* infection is typically characterized by attenuated inflammation and the transition of the parasite from the tachyzoite stage to the cyst stage [32]. During this stage of the disease, the brain becomes an important refuge for* T. gondii* and is usually associated with benign clinical features of disease [33]. Mice surviving early infection were monitored for additional 10 days and necropsied 25 d.p.i. to evaluate disease pathogenesis in later stages. The majority of tissues showed only minor signs of prior disease (Figure 3). However, we did observe a significant amount of neuroinflammation in the brains of all infected mice (Figure 6). Brain histopathology was evaluated by a board certified veterinary pathologist (S.C.O.) following H&E staining. Brain inflammation scores were generated based on assessments of the presence and amount of inflammatory cells within perivascular spaces and whether or not they extended into the adjacent parenchyma as well as the amounts of inflammatory cells present in the leptomeninges. The presence of* T. gondii* cysts within the tissue was also evaluated.* T. gondii* infection resulted in a significant increase in neuroinflammation in all of the infected mice at 25 d.p.i. compared to the mock treated animals (Figures 6(a) and 6(b)). However, the neuroinflammation observed in the* Casp11* ^−/−^ mice was significantly increased at this time point compared to the wild type and* Asc* ^−/−^ mice (Figures 6(a) and 6(b)). In the* Casp11* ^−/−^ mice, leukocytes were observed within perivascular spaces and often extended into the brain tissue (Figure 6(a)). We also observed significant differences in leptomeningitis following* T. gondii* infection (Figures 6(c) and 6(d)). Leptomeningitis refers to inflammation associated with the subarachnoid space in the brain and has been previously associated with* T. gondii* infection in humans [34, 35]. Leptomeningitis developed in all of the* T. gondii* infected mice. However, the condition was significantly increased in the* Casp11* ^−/−^ animals compared to the wild type and* Asc* ^−/−^ mice (Figures 6(c)-6(d)). The underlying cause of this highly serious presentation of toxoplasmosis in humans is unknown. However, the condition appears to occur more often in the context of immunosuppression or attenuated innate immune responses [34, 35].

### 3.7. Caspase-11 Attenuates* Toxoplasma gondii* Brain Cyst Burden

**Figure 7:**
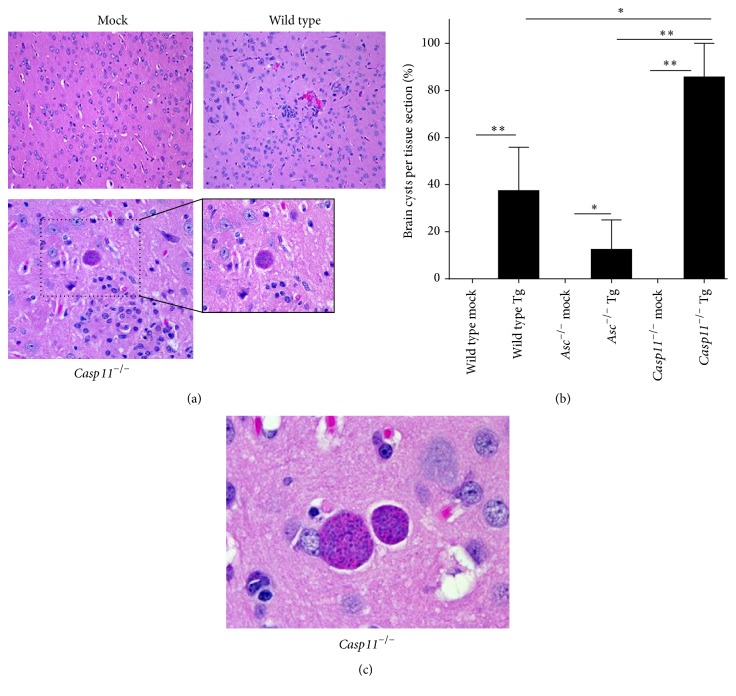
*Toxoplasma gondii* infection resulted in a greater brain tissue cyst burden in* Casp11* ^−/−^ mice during the chronic phase of disease. Wild type,* Casp11* ^−/−^, and* Asc* ^−/−^ mice were infected with 1,000* Toxoplasma gondii* Me49 tachyzoites via i.p. administration and brain tissue cyst formation was evaluated by histopathology 25 days after inoculation in surviving mice. (a) Histopathology assessments of H&E stained sections revealed tissue cyst formation in the brains from all genotypes infected with* T. gondii*. 20x magnification, 40x insert. (b) The presence of tissue cysts with the brain was assessed for each animal and the percent of animals with tissue cysts was determined. (c) High magnification image of a brain tissue cyst from a* Casp11* ^−/−^ mouse. 100x magnification. ^*∗*^ *p* < 0.05; ^*∗∗*^ *p* < 0.01. For all mock treated animals, *n* = 3. For all* T. gondii* inoculated animals, *n* = 7. Data shown are representative of 2 independent studies.

The formation of tissue cysts is the basis of* T. gondii* persistence in infected humans and animals. Indeed, the most frequent mechanism of primary infection is the ingestion of these tissue cysts [36]. In the brain,* T. gondii* generates latent cysts, which have been suggested to be associated with tachyzoite invasion of microglia, astrocytes, and neurons [37–41]. While these cysts tend to be considered asymptomatic in healthy individuals, reactivation in immunocompromised patients may result in fatal toxoplasmic encephalitis [42]. Cyst formation in the brain was evaluated by histopathology 15 and 25 d.p.i. (Figures 7(a)–7(c)). No brain cysts were identified in samples from mice harvested at 15 d.p.i. but they were present in mice harvested at 25 d.p.i. Brain cysts were detected less frequently in histopathology sections from wild type (37.5%) and* Asc* ^−/−^ (12.5%) mice compared with the* Casp11* ^−/−^ (85.7%) animals (Figure 7(b)). Increased cyst formation is a response of the pathogen to the stresses of the tissue environment including the host immune response (reviewed in [43]). Thus, this finding is consistent with our neuroinflammation observations in the* Casp11* ^−/−^ mice (Figure 6). Interestingly, these data are also consistent with prior studies that have noted a significant increase in brain cyst formation in caspase-1/caspase-11 double knockout mice and in* MyD88* ^−/−^ animals [16, 44]. The mechanisms underlying these observations are still undetermined. However, similar to the findings from the caspase-1/caspase-11 and MyD88 studies, our data reveals that caspase-11 also plays a critical role, either directly or indirectly, in attenuating brain cyst burden.

## 4. Discussion

Host resistance against intracellular pathogens, such as* T. gondii*, relies on a complex network of PRRs. The majority of studies to date have focused on TLR signaling pathways and have shown that resistance to* T. gondii* is driven by a diverse range of TLRs and is dependent upon the adaptor protein MyD88 [5–7, 44]. In addition to TLR signaling, members of the inflammasome forming NLR family have also been identified as critical mediators of host resistance to* T. gondii* [17]. Initial studies using human monocytes and genetic ablation of ASC revealed that IL-1*β* release was dependent upon canonical inflammasome components in this* in vitro* system [45]. Additional mechanistic studies revealed that canonical NLR inflammasome activation and IL-1*β* production in human monocytes were associated with the recognition of* T. gondii* protein GRA15 [45]. These studies supported previous observations, also in the human monocyte system, which revealed NLRP1 as the NLR responsible for inflammasome formation following* T. gondii* infection. Though we did not test individual NLRs as a part of our current study, our findings using BMDMs from* Asc* ^−/−^ mice largely support these prior observations [16, 17]. However, the direct sensing of GRA15, or any other known PAMP associated with* T. gondii*, by an NLR family member is yet to be established and the mechanism of inflammasome activation by this parasite is still quite unclear.

Beyond macrophage studies, the NLRP1 and NLRP3 inflammasomes have also been shown to significantly modulate* T. gondii* pathogenesis* in vivo* [17]. Mice lacking ASC, NLRP1, and NLRP3 are highly susceptible to parasite infection [17]. In these studies, the sensitivity to* T. gondii* appears to be associated with reduced systemic IL-18, rather than IL-1*β*, which allows increased parasite replication and reduced animal survival [17]. Mice deficient in IL-18 and IL-18 signaling were subsequently utilized to confirm this mechanism and the resultant findings revealed that these animals were also sensitive to* T. gondii* infection [17]. Our* in vivo* findings utilizing the* Asc* ^−/−^ mice are highly consistent with these prior observations and strongly imply that canonical inflammasome activation plays a vital role in promoting host resistance to* T. gondii*. While our overall conclusions support the prior studies, our current data does differ in the potential mechanism described. The apparent discrepancy between studies can be reconciled based on a variety of factors that can influence local and systemic cytokine levels following* T. gondii* infection in mice, including the infection dose/severity of disease, the timing of sample collection, the parasite strain, genetic modifications of the parental parasite strain, and the life cycle stage of the parasite at the time of sample collection [46].

It has recently come to light that studies of caspase-1 function have utilized* Casp1/11* ^−/−^ double knockout mice [17, 19]. Consistent with the loss of both canonical and noncanonical inflammasome signaling, macrophages from these animals also show ablated IL-1*β* [16, 17]. However, because both caspases are deficient in these animals, it has been difficult to discern a specific role for caspase-1 or caspase-11 in* T. gondii* pathogenesis. To better address this issue, we utilized* Casp11* ^−/−^ single knockout mice [19]. These animals have proven to be highly useful in characterizing canonical versus noncanonical inflammasome function following bacterial infection [19]. Here, we show that IL-1*β* production in* Casp11* ^−/−^ macrophages is significantly attenuated to levels which are comparable to those observed in* Asc* ^−/−^ macrophages (Figure 1). This is consistent with prior findings associated with caspase-11 function following LPS stimulation [19]. The current model of caspase-11 function focuses on its sensing of bacterial LPS, which has been shown to be a potent trigger of both canonical and noncanonical inflammasome signaling [47]. Extracellular LPS is recognized by TLR4 and serves as a priming signal for the canonical NLR inflammasome [48]. However, intracellular LPS appears to directly bind to and trigger caspase-11, resulting in canonical and noncanonical inflammasome activation in the cytoplasm [20]. Since* T. gondii* does not have LPS, this suggests that caspase-11 is associated with the recognition of some other aspect of this intracellular parasite to modulate IL-1*β* production. One possible mechanism could be associated withpotassium efflux, which is important to* T. gondii* egress from infected cells [49]. Recent studies using acute LPS exposure have suggested that potassium efflux can directly modulate caspase-11 activation of the NLRP3 inflammasome [50]. While this has not been directly evaluated in the context of* T. gondii* infection, this may be a possible mechanism underlying caspase-11 function.

Our data suggest that the loss of caspase-11 plays a protective role shortly after* T. gondii* exposure as* Casp11* ^−/−^ mice have improved survival and reduced local and systemic inflammation during earlier time points. These observations are consistent with the reduced levels of IL-1*β* and other proinflammatory cytokines, such as IL-6 (Figure 5). The pathogenesis is significantly different between the* Casp11* ^−/−^ and* Asc* ^−/−^ mice. Combined with the prior data related to ASC and caspase-1/11, these data suggest that caspase-1 and caspase-11 have distinct, nonredundant functions following* T. gondii* infection. It is possible that the canonical inflammasome modulates IL-18 and controls parasite replication, as previously reported [17], whereas caspase-11 and the noncanonical inflammasome modulate IL-1*β* and/or cell death, which act to increase local inflammation and minimize parasite migration to the brain. Supporting this hypothesis, in the* Casp11* ^−/−^ mice, we show attenuated local inflammation during acute infection yet with limited effects on tachyzoite burden in the local tissues (spleen and liver) (Figure 3). However, during the later stages of infection, the* Casp11* ^−/−^ mice present with greater neuroinflammation and cyst burden compared to the surviving wild type and* Asc* ^−/−^ animals (Figures 6 and 7). Similar data has been reported for* MyD88* ^−/−^ mice [44]. However, the mechanisms associated with the increased parasite migration and neuropathological effects were not fully discovered. Together, these data suggest that reduced local inflammation can result in improved morbidity and survival during early stages of disease, while creating an environment favorable for systemic expansion and ultimately increased brain localization.

The findings presented here suggest that caspase-11 function extends well beyond the currently characterized mechanisms associated with LPS recognition and acute bacterial infections. Our data show that caspase-11 is associated with increased inflammation during acute* T. gondii* infection, which results in attenuated neuroinflammation and reduced brain cyst burden during chronic phases of the disease. These data may suggest a role for caspase-11 in response to a currently undefined molecular pattern associated with* T. gondii* or may suggest that the noncanonical inflammasome is being activated by intracellular changes driven by the parasite, such as changes in potassium efflux. It is clear that additional mechanistic insight is needed to better define the interaction between these inflammasome pathways and parasite associated factors. Likewise, additional insight pertaining to the relationship between members of the TLR and NLR families in initiating the host immune response following* T. gondii* infection is necessary to better define the host-pathogen interactions. We anticipate that better defining the contribution of specific elements associated with the host innate immune response to this parasite will result in new therapeutic options and strategies to protect against this pervasive human pathogen.
